# Health Promoting Schools in Germany. Mapping the Implementation of Holistic Strategies to Tackle NCDs and Promote Health

**DOI:** 10.3390/ijerph18052623

**Published:** 2021-03-05

**Authors:** Kevin Dadaczynski, Thomas Hering

**Affiliations:** 1 Department of Nursing and Health Science, Fulda University of Applied Sciences, 36037 Fulda, Germany; 2 Centre for Applied Health Science, Leuphana University Lueneburg, 21337 Lueneburg, Germany; 3 Department of Applied Human Sciences, Magdeburg-Stendal University of Applied Sciences, 39676 Stendal, Germany; thomas.hering@h2.de

**Keywords:** health promoting school, implementation, non-communicable diseases, instrument development, capacity building, health and education

## Abstract

Noncommunicable diseases (NCDs) and their underlying risk factors are seen as major public health problems that threaten health and welfare systems worldwide. The holistic and resource oriented Health Promoting School (HPS) approach can serve as an appropriate framework for the prevention and control of NCDs. The paper aimed to map the implementation of HPS activities in German schools and to examine associations with potential influencing factors. A series of cross-sectional online studies including five federal states and 5006 school principals (40.2% males, 50.8% females) from primary and secondary public schools was conducted from 2013 to 2018. Principal component analysis (PCA) resulted in two factors of HPS implementation (F1: concrete HPS action, F2: capacity building for HPS). Comparing both factors, a lower implementation level could be identified for HPS capacity building with lowest mean values found for regular teacher training and intersectoral collaboration. Multiple binary regression analyses revealed significant associations between low HPS implementation and male gender (OR: 1.36 to 1.42), younger age (OR: 1.47 to 1.90), secondary school (OR: 1.78 to 3.13) and federal state (Lower Saxony = OR: 1.27 to 1.45; Schleswig-Holstein = OR: 1.95 to 2.46). Moreover, low access to resources, decision-latitude and perceived educational benefits were independently associated with both factors of HPS implementation. Based on the results of this study, there is a need to support schools in their capacity building for health (e.g., regular teacher training, cooperation with local health services). Moreover, considering the core mission of schools, more evidence of the educational impact of health promotion and its translation into the language of education is needed for secondary schools in particular.

## 1. Introduction

According to the World Health Organization (WHO) [1], about 41 million people worldwide died of noncommunicable diseases (NCDs) in 2016, which corresponds to 71% of all deaths. In this context, cardiovascular diseases, cancers, chronic respiratory diseases, diabetes, and mental health conditions are seen as the main types of NCD. The Global Burden of Disease (GBD) study is one of the most comprehensive studies worldwide, including 369 diseases and injuries and 87 risk factors for 204 countries. Recent results from the GBD 2019 identified six NCDs among the top 10 causes of disease burden across all age groups [2]. While a global decline of several risk factors could be observed from 2010 to 2019 (e.g., including unsafe water, sanitation, tobacco smoking) other modifiable risk factors such as drug use or high body-mass index increased [3]. Among the top risks for attributable deaths in 2019 were high systolic blood pressure, dietary risks and tobacco smoking [3].

To tackle NCDs and their underlying risk factors, the World Health Organization (WHO) has developed a road map for 2013 to 2020 including six objectives and nine voluntary global targets [4]. The creation of health-promoting environments such as schools is emphasized in objective 3 but also in other policy documents such as the Paris declaration on ‘partnerships for the health and well-being of our young and future generations’ [5]. Schools have long been regarded as important settings for health promotion and prevention for several reasons: first, as the individual determinants of NCDs (e.g., knowledge, health literacy, attitude, behaviour) are already established in the early phases of the life course, health promotion and prevention activities should start as early as possible in childhood and adolescence. The most recent results from the German Health Behaviour in School-aged Children (HBSC) study show that only 10.0% of girls and 17% of boys meet the World Health Organization’s (WHO) physical activity recommendations, while the percentage of daily fruit and vegetable consumption varies between 23% (vegetable intake boys) and 42% (fruit intake girls) [6]. In addition, the 30-day prevalence of tobacco use is 6.7% and of alcohol use is 23.6% [7]. With regard to the personal competencies to find, understand and use health information, HBSC results revealed a low and moderate health literacy level for 87% of German young people [8]. Second, schools provide young people with an inclusive and equitable access to education as a key determinant of health regardless of their socioeconomic, cultural or political background. This is especially important as most findings presented above follow a social gradient with positive associations between health behaviour, health literacy and family affluence [6,7,8]. Third, schools are not merely access points to children and young people, but also have an influence on health and health behaviour through their structures, conditions and processes (e.g., physical activity friendly school yards, food service environment [9,10]). Fourth, research findings from recent years suggest that determinants of NCDs such as physical inactivity [11] or overweight [12], but also mental health problems [13], can have adverse effects on school performance (e.g., grades, test scores) and thus can compromise the core mission of schools.

Compared to measures, that focus on single health determinants and target groups, it is argued that holistic intervention approaches on school health promotion are more promising as they move beyond individual behaviour by also addressing the physical and social environment and take into account all members of the school and the wider school-community. Favored by the World Health Organization [14] the Health Promoting School (HPS) can be characterized as “[…] a school constantly strengthening its capacity as a healthy setting for living, learning and working“ (p. 357, [15]). In contrast to external (often pre-packaged) interventions brought into the school with the intention of being implemented with high fidelity, the HPS strives for changes that are initiated by the schools themselves according to their specific needs. This complex approach requires a participatory process in which measures are implemented on the basis of needs planning and the empowerment of all people involved [16,17]. As highlighted in the Moscow Declaration, the HPS offers a resource-based intervention framework to combat NCDs and thus differs from the traditional top-down and risk factor-oriented approaches that dominate in NCD prevention. It shifts the focus from an exclusive perspective on individual behaviour to a comprehensive socioecological perspective, by including environmental determinants and by considering people within and outside the school as agents of healthy change processes [18]. Despite their international popularity, there is no common understanding of the core elements and main fields of action of holistic approaches to school health promotion. Based on a retrospective correlation study, Lee et al. [19] identified 20 core indicators among six key areas of Health Promoting Schools (school environment (physical and social), school policies on health, community links, action competencies, school health care and promotion services) which have been further substantiated by a recent scoping review [20]. Similarly, the Schools for Health in Europe (SHE) network has defined six core components (healthy school policies, physical and social environment, individual health skills, community links and health services) in order to seek to achieve a whole-school approach. Moreover, eight European Standards have been proposed covering 15 areas to facilitate the implementation of Health Promoting Schools [21]. Next to the six key core components these also include the organizational structure, school leadership and health literacy. In turn, the US American Whole School, Whole Community, Whole Child (WSCC) model calls for intersectoral alignment and emphasizes communities and their role in supporting schools. It includes 10 components addressing different topics (e.g., nutrition, physical activity), structures (physical environment) and community actions (e.g., counselling, psychological and social services, family engagement) [22]. In Germany, a conceptual distinction is made between the Health Promoting School approach and the good healthy school approach. While the HPS is understood as a school development process that aims at maintaining and promoting the health of all people in the school, the good healthy school approach focuses on promoting educational quality through health [23]. Both concepts rely on four fields of action (teaching/learning; school culture; services & cooperation; management strategies) and are rooted in basic principles such as participation, empowerment, or networking. Although there remains a paucity of evidence, study findings indicate that holistic school approaches can have a positive impact on several determinants of NCDs such as body composition (e.g., BMI) [24,25,26], healthy eating practices (e.g., fruit and vegetable consumption) [25,26,27,28], physical activity and fitness [25,26,27], or mental health outcomes (e.g., social–emotional competencies and aggressive behaviour) [25,27,29].

Despite the fact that the HPS approach is considered as a key strategy in public health research, policy and practice, it is surprising that so far relatively little is known about the extent to which schools actually implement activities relating to school health promotion and prevention in practice. In their study, Vilaça et al. [30] collected data on various aspects of HPS implementation from the national coordinators from the member countries of the Schools for Health in Europe (SHE) network (*n* = 24). Results revealed that more than half of respondents (58%) reported that their country has a national HPS strategy that is partially integrated in national education and public health policies. With regard to the topics addressed, most national HPS strategies (87.5%) include more than nine health topics with physical activity, healthy eating, and mental health most frequently mentioned. So far, there have been hardly any attempts to systematize and map health promotion activities in schools in Germany. There are several German intervention databases available such as the good practice database of the national Cooperation-Network (CN) ‘Equity in Health’ [31] which lists approximately 320 school-based interventions. However, not all of these interventions take a whole school approach to health promotion, but rather follow a very narrow approach, e.g., by focusing on individual level behaviour and its determinants alone (health promotion in settings) [32]. Preliminary evidence from the German Health Behaviour in School-Aged Children (HBSC) 2009/2010 study revealed that more than half of all schools surveyed (53.1%) implemented health related activities on a regular basis [33]. The topics most frequently addressed were prevention of addictive behaviour (82%), the promotion of physical activity/sport (81.3%) and communication/conflict resolution (77.7%). Due to national health policies, statutory health insurance is required to offer health-promoting activities in these settings and to report on them annually. The most recent prevention report indicates that in 2019 over 12,000 primary schools and more than 10,000 secondary schools have been reached. The percentage of interventions addressing combined strategies (individual and environmental level) was 49% for primary schools and 79% for secondary schools [34]. However, the uptake of those interventions by schools and their systematic integration in regular school routines remain unknown.

As pointed out by Herlitz et al. [35] in their recent systematic review with 24 studies of 18 interventions, no school intervention was sustained in its entirety after external funding or support ended and no relationship could be found between intervention effectiveness and sustainability. However, the authors could identify a number of barriers and facilitators including availability of resources and focus on educational outcomes. The latter has been described as a major barrier as schools often perceive health promoting measures as supplanting time actually needed for teaching core subjects such as math or reading [36,37]. It may be argued that schools that consider the educational benefits of health promotion activities to be high are also more likely to implement such interventions. Sufficient resource allocation was identified as another influencing factor in a number of studies. Qualitative findings from the US [38] and Finland [39], for example, revealed a lack of finances and time allocated to health promotion as stumbling blocks for educators in their implementation efforts. Finally, there is growing evidence pointing to the specific role of leadership in implementing holistic HPS intervention strategies. Amongst others, these include visionary or transformative leadership behaviour, competencies, or continuous support [40,41,42]. However, work-related resources of school leaders and their relevance for the implementation of health promotion have so far barely been the subject of research. Decision latitude can be regarded as a job characteristic that reflects an individual’s ability to make decisions about his or her own job (decision authority) and/or the degree to which the job involves various tasks and requires the development of new abilities (skill discretion) [43]. Low decision latitude has been identified as a risk factor for physical and mental health [44,45] and theoretical work suggests decision latitude as a work factor contributing to an organizationally health environment [46]. It can therefore be assumed that a high level of decision latitude increases the likelihood of implementing school health promotion activities.

Drawing on the limited research findings described above, this study aimed to map the implementation of HPS activities in German schools and to examine associations with potential influencing factors. The following research questions guided this study:

1. What is the state of HPS implementation in German schools?
2. Does HPS implementation differ with regard to demographic and school characteristics?
3. What are the associations between HPS implementation and demographic and work characteristics and further influencing factors (i.e., resource availability, decision latitude, and perceived link between health and education)?

## 2. Materials and Methods

### 2.1. Study Design and Study Population

For this paper, we used data from a German cross-sectional school principal survey, that started in 2012 as an ongoing empirical project. Based on limited evidence on school principals in school health promotion, the aim of the survey was to investigate the working conditions and the health situation of this occupational group, and to examine the role of school principals in HPS implementation. First started in North Rhine-Westphalia in late 2012/early 2013, the study was also conducted in the German federal states of Berlin, Lower Saxony, Schleswig-Holstein, and Hesse from 2014 to 2018. To ensure comparability, a core questionnaire was used in each study. All school principals and members of the school management board (e.g., vice-principals) from public primary and secondary schools were eligible to participate in this survey. Following ethical approval by the Ministry of Education of each participating federal state, potential respondents were invited to participate in this survey. All communication and invitation efforts were organized in cooperation with the school principal association in the respective federal state, which had access to the email addresses of all schools. The communication also included two reminders during the data collection phase, as well as information provision via the websites and newsletter services of the school principal associations. The survey was administered electronically using the Enterprise Feedback Suite (EFS) survey tool by Questback (Cologne/Germany). Participation was voluntary, and anonymity was assured. Upon entering the online survey site, participants were presented with information regarding the aims and the background of the study including a consent box at the bottom of the page.

After a plausibility check, data adjustment, and merging of the data from the individual surveys, the final sample contained a total of *n* = 5006 respondents. With approximately 41%, the majority came from North Rhine-Westphalia, followed by respondents from Lower Saxony (26.7%) and Schleswig-Holstein (14.3%, see Table 1). In terms of demographic characteristics, females and respondents aged 46 to 60 years were most frequently represented with about 60% each. Regarding type of school, 45% of respondents belonged to a primary school, while 7% worked at a vocational school during the time of data collection.

### 2.2. Measures

The implementation of the HPS served as main outcome in this study. The starting point for measuring the implementation status of health promotion in schools was a scale with eight items developed and tested in a previous German study on school health management (e.g., “At our school, maintaining and promoting the health of all members plays an important role.”) [47]. As this scale did not reflect all dimensions of a holistic approach to school health promotion (e.g., school curriculum, school environment, school-community links, multi-target group orientation [16,23,25]) we extended this instrument by six self-formulated items (e.g., “Our school collaborates with external institutions in the implementation of health-promoting activities.”). All items could be answered on a 4-point Likert-type scale (1 = strongly disagree, 4 = strongly agree). After developing additional items within the research group, the extended scale was pretested with selected school principals and teachers (*n* = 6). Based on their feedback, slight changes in the formulations were made.

Access to resources, decision latitude, and the perceived link between health and education were included as explanatory variables. Access to resources was measured using three items developed by Spreitzer [48]. Her study was guided by the assumption that access to critical organizational resources (e.g., budget, staff, information) result in enthusiastic and engaged individuals with increased ownership and sense of empowerment to master work tasks. While the original scale employed a 7-point Likert scale, we used an adapted 5-point Likert scale ranging from 1 (not true) to 5 (completely true). One example item was “I can obtain the resources necessary to support new ideas”. Cronbach’s α for this variable was 0.78. For binary regression analysis, we dichotomized this variable (0 = high access to resources, 1 = low access to resources) based on median-split.

Decision latitude has been used as an organizational resource that is reflected in an individual’s ability to make decisions about his or her own job. Derived from the German questionnaire on the work situation at schools [49], decision latitude was operationalized with four items that could be answered on a 5-point Likert scale (1 = not true at all, 5 = completely true). One example item was “Overall, the work offers many opportunities to make own decisions”. Due to a low internal consistency, one item had to be excluded. Cronbach α for the remaining three items was acceptable (0.70). Using median-split this variable was dichotomized into a group with low (=1) and high (=0) decision latitude.

To assess the perceived educational benefits of health promoting activities at school, a self-developed single item was used: “If health promotion and prevention activities were continuously implemented at my school, I believe it would have a positive impact on students’ academic performance”. Response options include a 4-point Likert scale (1 = very unlikely, 4 = very likely). Again, median-split was used to create two groups with low (=1) and high (=0) perceived educational benefits.

We used gender (male, female) and age (≤45 years, 46–60 years, >60 years) as sociodemographic variables. School characteristics included type of school (primary school, secondary school, schools for children with special educational needs, vocational schools and others) and federal state (Berlin, Hesse, Lower Saxony, North Rhine-Westphalia, Schleswig-Holstein).

All instruments used for this study can be found in Table S1.

### 2.3. Statistical Analyses

As a first step, a principal component analysis (PCA) with orthogonal varimax rotation was performed to investigate the correlative structure of the instrument on HPS implementation. In an initial PCA, all 14 items of the instrument were included (see Table S2) and their fit was tested using Kaiser-Meyer-Olkin criterion (KMO > 0.6, moderate or better) and Bartlett’s test for sphericity (*p* < 0.05) [41]. All factors based on the Scree-test with eigenvalues > 1 were extracted. After visual assessment of the content relevance, items with communalities ≤ 0.45 and loadings < 0.50 were removed [50,51]. Another PCA was calculated with the remaining items, which led to the final solution. The reliability of the extracted factors was assessed by their internal consistency (Cronbach α).

In a second analytical step, we used median split to dichotomize the two HPS implementation factors identified in the PCA (low = 1 vs. high = 0). Next to univariate analysis (mean values, standard deviations), cross tabulation and chi-square tests (χ^2^) were performed to test the statistical significance of the association between categorical data (i.e., the two levels of each HPS implementation factor with demographic and school characteristics).

This association was further explored in a third step through logistic regression modelling, to analyze the contribution of demographic and school characteristics and of further influencing factors on HPS implementation. Separate models were calculated for each HPS implementation factor by odds ratio (OR) and its respective 95% confidence interval (95% CI). Based on research results reported above [24,25,26,27,28,36], high levels of resource availability, decision latitude and high perceived educational benefits were assumed to be positively associated with HPS implementation and hence served as reference categories. Moreover, due to preliminary evidence from Germany which indicated a higher school health promotion activity for primary schools [52], we used this type of school as reference. With regard to gender and age, the reference categories were formed based on the assumption that female and older respondents show a greater awareness of health-related topics and are therefore positively associated with a higher HPS implementation.

The estimated fit of the regression models was provided by Nagelkerke’s R squared (*R*^2^). For all analyses *p*-values < 0.05 were considered statistically significant. Statistical analyses were performed using IBM SPSS version 23 (IBM, New York, NY, USA).

## 3. Results

### 3.1. Factorial Structure of the HPS Implementation Instrument

The correlation matrix of the 14 items on HPS implementation is suitable for a PCA (KMO = 0.93, Bartlett-Test χ^2^ = 28426, *p* < 0.001). Two factors with an eigenvalue > 1 were extracted, explaining 53.8% of the total variance (Table 2). Each factor is composed of 7 items. The factor loadings for factor 1 are between 0.46 (item 9: Systematic improvement of the work situation) and 0.77 (item 1: Regular further training on health-related topics). For factor 2, the loadings range from 0.57 (item 7: Teacher support in dealing with stressful situations) to 0.73 (item 5: Health promoting working and learning conditions). While the items of factor 1 point to ‘capacity building for HPS’ (e.g., regular further training on health related topics, collaboration with external institutions), factor 2 contains items that focus more on ‘concrete HPS actions’ (e.g., creating working and learning conditions, development of health-promoting behaviors).

However, not all items met the predefined criteria to be retained in the final solution. Items No 9, 10, 13, and 14 were not adequately explained by the extracted factors (communalities ≤ 0.45) and hence were excluded. Furthermore, item 9 loaded almost equally on both factors with a factor loading < 0.50. The content analysis also revealed overlaps with other items that justify exclusion. The correlation matrix of the remaining 10 items also proved to be suitable for a principal component factor analysis (KMO = 0.92, Bartlett-Test χ2 = 22889, *p* < 0.001). Two factors with an eigenvalue > 1 were extracted, explaining 63.2% of the total variance (Table 3). Each factor of the final solution comprises five items. The factor loadings of factor 1 range from 0.58 (item 7: Teacher support in dealing with stressful situations) to 0.81 (item 4: Importance of health promotion for all members in the school). Factor 2 loadings range from 0.60 (item 2: Health goals in the school’s mission statement) to 0.82 (item 12: Regular further training on health-related topics). As in the first PCA (but in reversed order), the factors of the final solution can be summarized as ‘concrete HPS actions’ (F1) and ‘capacity building for HPS’ (F2).

### 3.2. Level of HPS Implementation

Compared to the dimension ‘capacity building for HPS’ (*M* = 2.56, SD = 0.71), a significant higher level of HPS implementation could be found for the dimension ‘concrete HPS action’ (*M* = 2.87, SD = 0.60, *p* < 0.001). On the level of single items, results showed highest mean values for supporting students in their health promoting behaviors (item 6, *M* = 3.13, SD = 0.69) and for the integration of health promotion in school developmental groups (item 1, *M* = 2.91, SD = 0.92). In contrast, lowest mean values could be observed for regular further training on health topics (item 12, *M* = 2.19, SD = 0.83) and for collaboration with external institutions (item 8, *M* = 2.41, SD = 1.01).

Stratified by sociodemographic characteristics, chi square tests revealed significant gender and age differences. Female respondents and those aged > 60 years reported a higher level of concrete HPS action (*p* < 0.001) and capacity building (gender: *p* < 0.001, age: *p* < 0.01, Table 4). With regard to type of school, respondents from primary schools (59%) and from schools for children with special educational needs (61%) more often reported higher levels of concrete HPS action (*p* > 0.001). On the other hand, highest frequencies for a high HPS capacity building were found for vocational schools (53%) and primary schools (47%). Across both dimensions, secondary schools most frequently indicated a low HPS implementation (concrete HPS action: 68%, capacity building for HPS: 68.5%). Stratified by federal state, descriptive results showed significant differences with respondents from Schleswig-Holstein more often reporting a low level of concrete HPS implementation. In turn, a high level of concrete HPS action and capacity building could be most frequently observed for schools from North Rhine-Westphalia and Hesse (see Figure S1).

Although excluded in the final PCA solution with 10 items, collaboration with parents and the creation of a health promoting school environment represent important aspects of holistic approaches to school health promotion with mean values ranging between *M* = 2.7 to 2.8 (see S1). Stratified by sociodemographic and school characteristics, results show similar patterns with higher levels of implementation found for female principals (*p* < 0.001) and primary school principals (*p* < 0.001). While lowest level of collaboration with parents could be found in the federal state of Berlin (*M* = 2.67, *p* < 0.001), school principals from the federal states of Hesse (*M* = 2.52) and Schleswig-Holstein (*M* = 2.53) report lowest values for the creation of health promoting environments (*p* < 0.001).

### 3.3. Prediction of HPS Implementation

The results of the multiple binary regression analysis are displayed in Table 5. Regarding factor 1: ‘concrete HPS action’ findings revealed significant associations with all demographic and school characteristics. Male respondents and those from the youngest age group (≤45 years) reported a lower level of concrete action (male = OR: 1.36, 95% CI: 1.18–1.56; ≤45 years = OR: 1.90, 95% CI: 1.55–2.34). Compared to primary schools, secondary schools had a 3.13-fold (95% CI: 2.68–3.64) and vocational schools had a 2.20-fold (95% CI: 1.69–2.87) increased risk of low concrete HPS action. Moreover, belonging to the federal states of Lower Saxony (OR: 1.45, 95% CI: 1.24–1.70) or Schleswig-Holstein (OR: 2.46, 95% CI: 2.01–3.01) was associated with a higher probability of a low level of concrete HPS action. Significant associations could also be observed for resource availability, decision latitude, and perceived educational benefits. For respondents reporting a low access to resources, we observed a 1.46-fold increased risk of low HPS implementation for factor 1 (95% CI: 1.27–1.67). The same could be shown for low level of decision latitude (OR: 1.55, 95% CI: 1.34–1.79) and low perceived educational benefits of health promotion (OR: 1.65, 95% CI: 1.36–2.01). A Nagelkerke’s R^2^ of 0.16, suggests that 16 percent of the variation between the two groups could be explained by the variables included in the regression analysis.

For factor 2: ‘capacity building for HPS’, the findings point in a similar direction. Male sex and younger age (≤45 years) were each associated with more than a 1.4-fold increased risk of low HPS capacity building (male = OR: 1.42, 95% CI: 1.24–1.63; ≤45 years = OR: 1.47, 95% CI: 1.20–1.79). While belonging to a secondary school was associated with an increased risk of reporting a lower level of capacity building (OR: 1.78, 95% CI: 1.54–2.07), the opposite was found for vocational schools (OR: 0.73, 95% CI: 0.56–0.94). With regard to geographical location, a significant increased risk for low capacity building could be observed for Lower Saxony (OR: 1.27, 95% CI: 1.08–1.48), Berlin (OR: 1.90, 95% CI: 1.38–2.61) and Schleswig-Holstein (OR: 1.95, 95% CI: 1.59–2.38). All remaining variables were also positively associated with the dependent variable with odds ratios ranging from OR = 1.29 (access to resources) to OR = 1.69 (perceived educational benefits). The variation between the two groups (i.e., high and low strategic capacity building for HPS), explained by the explanatory variables in the regression models, was R^2^ = 0.09.

## 4. Discussion

The present work aimed to map the implementation of Health Promoting Schools (HPS) in Germany and to examine associations with demographic, work and other influencing factors. To date, only a few instruments exist that capture holistic school health promotion strategies across different school types with sufficient psychometric quality [53]. In order to take the German context into account, a new instrument was developed and field-tested. With its 10 items capturing two dimensions (‘concrete HPS action’ and ‘capacity building for HPS’), the instrument is efficient and suitable for use in larger studies. However, the brevity of the instrument also resulted in the fact that not all aspects and elements of the HPS have been covered in detail. Although essential for holistic approaches to school health promotion, the items on cooperation with parents and on the creation of the school environment had to be excluded from the final solution of the principal component analysis. Against this background, further research activities should be undertaken to further refine this instrument on HPS implementation.

Results from the single surveys included in this analysis show that schools pursue hands-on activities on health promotion more extensively than activities on capacity building. Although it can be regarded as positive that schools are implementing practical measures to promote health, research attaches a great importance to strategic and systemic planning including the necessary capacities for sustainable anchoring of health promotion in schools [37,40]. The lowest level of implementation could be found for regular further training on health-related topics and for collaboration with external stakeholders (both belonging to the dimension ‘capacity building for HPS’). Research suggests that continuous training and professional skills are important enablers for supporting the implementation of HPS [54,55]. However, in German teacher education, health promotion and prevention are not obligatorily anchored and there are only a few universities that have systematically integrated health into their initial teacher training programs. Although there are now many further teacher training offers on health related topics, these vary from one federal state to another and are also not compulsory. Even though an overview of the available training courses is still lacking, a situation comparable to Austria can be assumed due to similar education systems. Based on a content analysis of Austrian university curricula and modules, Flaschberger [56] identified a focus on individual behavior and on one-off seminars without follow-up. Hence, the course offers should therefore be more oriented towards the setting approach and be aligned with the core competences for health educators developed by Moynihan et al. [57], including knowledge, skills and attitudes. The rather low level of cooperation between schools and health-related stakeholders are supported by findings from Ireland [58] and could be due to various reasons. On the one hand, health is not sufficiently anchored in the school laws of the federal states, which could lead to an underrepresentation of cooperation with health-related stakeholders. Simultaneously, with few exceptions, intersectoral cooperation in German health promotion has only been advanced in recent years [59,60]. The Dutch Diagnosis of Sustainable Collaboration (DISC) model provides an innovative conceptual framework whose factors (external factors, change management, context, project management, stakeholders’ support) have been shown to be significant predictors of HPS implementation [61]. The barriers and success factors identified in this and similar research should be used to stimulate partnerships between schools and the local community in the future.

Stratified by sociodemographic variables, female and older respondents are found to report a higher HPS implementation for their schools. As suggested by Tannenbaum et al. [62], sex and gender are crucial to consider when it comes to the uptake and implementation of any health intervention. The reasons for this can be manifold and include, among other things, that women are more sensitive to health-related issues [47] and make more frequent use of health-related services [63]. Moreover, research findings indicate that woman tend to apply a transformational leadership style more often while men tend to use other aspects of transactional and laissez-faire leadership behavior [64]. With regard to age, it can also be assumed that older school principals’ have more work experience and fewer multiple stresses (e.g., work-life balance) compared to younger principals and attach greater importance to health-related issues. However, the extent to which these factors have a positive effect on HPS implementation should be investigated in further qualitative studies.

School type and federal state served as school characteristics and, in line with our assumption, primary schools reported higher levels of concrete HPS action while secondary schools scored lowest. It could be argued that secondary schools are characterized by a stronger subject orientation compared to primary schools, which creates greater pressure to achieve high educational outcomes among their students. In turn, primary schools (and schools for children with special educational needs) are more focused on the holistic development of their pupils, which gives them more opportunities to implement health-promoting activities. Geographical location also served as significant predictor with respondents from Schleswig-Holstein most frequently reporting a low level of HPS implementation. This finding is in line with previous results from qualitative research. In their analysis of German school laws Niehues et al. [65] identified federal states who placed less emphasis on school health promotion in their legislation. Even 15 years later, there are federal states, where health promotion is more strongly anchored in the school laws and where a number of complex state-wide projects have been realized with a broad network of cooperating partners (e.g., North Rhine-Westphalia). These results highlight the importance of the context, which according to the Context and Implementation of Complex Interventions (CICI) framework can be defined as “[…] a set of characteristics and circumstances that consist of active and unique factors, within which the implementation is embedded” (p. 6, [66]). It comprises seven domains (i.e., geographical, epidemiological, socio-cultural, socio-economic, ethical, legal and political factors) which interact with implementation and setting as the other two dimensions of the framework. As suggested by the authors, the framework can serve as a basis when examining complex public health interventions and their implementation and effectiveness.

In line with the context domain, the results of our study also indicated that respondents who perceive educational benefits of health promotion activities as high also report a higher level of HPS implementation. As schools are primarily legitimized as educational organizations, the connection between health and education has been taken up for about 15 years in the German approach of the good and healthy school. Compared to the HPS, the good and healthy school applies health promoting interventions in order to support the educational mission of schools and to strengthen educational outcomes and school quality [23]. Hence, health promotion is regarded as an input factor for education and not merely for health outcomes. Although the causal link between health and education has been increasingly examined in recent years, public health research has failed to demonstrate the educational impact of HPS related interventions [25]. Most recent evidence from Wales suggest that schools with more health improvement activities showed better attendance and English, Math and Science attainment with beneficial outcomes among more deprived schools [67]. Given the link between perceived educational benefits and HPS implementation, more evidence and its translation into the language of education is needed for secondary schools in particular.

While the relationship between resource availability and HPS implementation confirms the findings of previous studies [35,38,39], the results on the role of decision latitude are novel. According to the demand-control model, a high ability to make decisions on the job and work tasks is seen as an important ingredient for an active job [43]. Research has identified decision latitude and autonomy not only as a predictor for job satisfaction and health among school leaders [45], but also as important contributing factor for work engagement [68], organizational commitment [69] and organizational change [42]. Strengthening decision-latitude and autonomy could therefore serve two goals at the same time, the health of educators and the implementation of health-promoting change processes in schools.

To our knowledge, this is the first study mapping the implementation of holistic strategies on school health promotion across German federal states. However, some limitations need to be taken into account: first, although the sample size is fairly high, the findings are not generalizable due to its convenience sampling method. Second, online surveys always include the potential risk of excluding people with limited access to digital devices or with limited technical skills. However, we consider the risk to be low as most school principals have their own computer with access to the internet. Third, the cross-sectional nature of this study does not allow us to draw any causal conclusions. Fourth, the biggest limitation concerns the time lag of five years between the first study in North Rhine-Westphalia and the latest in Hesse. There might be a number of time sensitive factors that account for the differences in HPS implementation between the federal states. One of them is the national Prevention Act, that has been introduced by the German Federal Ministry of Health in 2015. It aims to strengthen health promotion and primary prevention in settings (e.g., schools, day care settings), to improve coordination between stakeholders and to reduce health inequalities. In order to achieve the goals, statutory health insurances are obliged to increase their annual expenditure on health promotion activities and the latest prevention report indicates a significant increase in health promoting activities since 2016 [34]. Therefore, further studies are needed to examine the current state of HPS implementation after the introduction of the Prevention Act. Finally, school leaders were the only source of information. As health promotion in schools is often implemented by teachers, future consideration of this group (e.g., teachers) would contribute to a more validated assessment of the state of HPS implementation.

## 5. Conclusions

In contrast to a top-down and disease-oriented approach, the HPS offers a resource-oriented intervention approach based on health-promoting values and pillars in tackling NCDs [18]. The study findings provide new insights into the implementation of HPS in Germany. Compared to hands-on activities, there is a need to support schools in their capacity building for health. Most specifically, this includes the provision of regular teacher training on single health topics and on how to design health promoting change processes within the school. Moreover, systematic activities that stimulate meaningful collaboration between schools and local health services are needed. HPS implementation is associated with contextual characteristics such as type of school and federal state (e.g., policies and structures). Considering the core mission of schools, more evidence on the impact of health promoting interventions on educational outcomes is needed. Translated into the language of education, this could lead to an integrative perception of health as a resource for education with positive effects on resource allocation and an improvement in the implementation of health promotion in schools. In order to achieve direct health benefits and a broader implementation of school health promotion activities, decision-latitude and autonomy should be improved.

## Figures and Tables

**Table 1:** Characteristics of study participants.

Item	Category	Frequency (*n*)	Percentage (%)
Gender	Male	2014	40.2
	Female	2991	59.8
Age	≤45 years	1025	20.5
	46 to 60 years	3004	60.0
	>60 years	976	19.5
Type of school	Primary school	2220	44.9
	Secondary school	1793	36.3
	Schools for children with special needs	564	11.4
	Vocational school	336	6.8
	Other	28	0.6
Federal State	Berlin	237	4.7
	Hesse	680	13.6
	North Rhine-Westphalia	2039	40.7
	Lower Saxony	1336	26.7
	Schleswig-Holstein	714	14.3

**Table 2:** Initial and final principal component analysis (PCA)-solution of the Health Promoting School (HPS) implementation instrument.

		Initial 14-Item Solution	Final 10-Item Solution
Kaiser-Meyer-Olkin Criterion (KMO)		0.93	0.92
Bartlett-test		χ^2^ = 28426, *p* < 0.001	χ^2^ = 22889, *p* < 0.001
Factor intercorrelation		0.68, *p* < 0.001	0.68, *p* < 0.001
Items	Factor 1 Factor 2	1, 2, 8, 9, 10, 11, 12 3, 4, 5, 6, 7, 13, 14	3, 4, 5, 6, 7 1, 2, 8, 11, 12
Eigenvalue	Factor 1 Factor 2	6.331 1.210	5.304 1.016
Explained variance	Factor 1 Factor 2	45.2% 8.6%	53.0% 10.2%
Communalities (>0.45)	Factor 1 Factor 2	0.38 (item 9) to 0.66 (item 5)	0.49 (item 7) to 0.76 (item 4)
Factor loadings (>0.50)	Factor 1 Factor 2	0.459 to 0.768 0.572 to 0.728	0.575 to 0.813 0.604 to 0.816
Reliability (Cronbach α)	Factor 1 Factor 2	0.84 0.85	0.83 0.87

**Table 3:** Factor loadings of the final HPS implementation instrument with 10 items.

Item No	Description	Factor 1 (α = 0.83)	Factor 2 (α = 0.87)
Item 4	At our school, maintaining and promoting the health of all members plays an important role.	**0.813**	0.307
Item 5	Health-promoting aspects play an important role in the creation of working and learning conditions at our school.	**0.810**	0.276
Item 3	At our school, health plays an important role in the organization of lessons.	**0.778**	0.324
Item 6	At our school, pupils are supported in the development of health-promoting behaviors.	**0.748**	0.216
Item 7	At our school, teachers are supported to deal with stressful situations more effectively.	**0.575**	0.398
Item 12	Further training on health-related topics take place regularly at our school.	0.200	**0.816**
Item 11	At our school, teachers are made aware of health-related topics such as exercise or self-management.	0.307	**0.735**
Item 8	Our school collaborates with external institutions in the implementation of health-promoting activities.	0.251	**0.727**
Item 1	Health promotion is a topic in our school development group.	0.310	**0.691**
Item 2	Health promotion and health goals are anchored in the mission statement and program of our school.	0.398	**0.604**
Explained variance (total 63.2%)		53.0%	10.2%

Notes. Bold values indicate the assignment of the items to the factors.

**Table 4:** HPS implementation stratified by sociodemographic and work characteristics.

	Concrete HPS Action		HPS Capacity Building	
	Low	High	Low	High
	% (*n*)	% (*n*)	% (*n*)	% (*n*)
Gender (*n* = 4741)	*χ*^2^ (df = 1) = 61.256, *p* < 0.001		*χ*^2^ (df = 1) = 49.516, *p* < 0.001	
Male	58.6 (1117)	41.4 (789)	64.7 (1239)	35.3 (1962)
Female	47.0 (1333)	53.0 (1505)	54.5 (1540)	45.5 (1287)
Age (*n* = 4741)	*χ*^2^ (df = 2) = 26.532, *p* < 0.001		*χ*^2^ (df = 2) = 11.316, *p* < 0.01	
≤45 years	57.2 (549)	42.8 (411)	63.1 (610)	36.9 (356)
46 to 60 years	51.9 (1481)	48.1 (1374)	57.9 (1652)	42.1 (1200)
>60 years	45.4 (420)	54.6 (506)	56.0 (517)	44.0 (406)
Type of school (*n* = 4677)	*χ*^2^ (df = 4) = 305.369, *p* < 0.001		*χ*^2^ (df = 4) = 114.815, *p* < 0.001	
Primary school	41.1 (865)	58.9 (1242)	52.8 (1110)	47.2 (992)
Secondary school	67.6 (1141)	32.4 (548)	68.5 (1157)	31.5 (533)
School f. child. with special educ. needs	39.1 (209)	60.9 (326)	58.6 (316)	41.4 (223)
Vocational school	58.1 (187)	41.9 (135)	47.2 (151)	52.8 (169)
Others	45.8 (11)	54.2 (13)	66.7 (18)	33.3 (9)
Federal state (*n* = 4741)	*χ*^2^ (df = 4) = 105.587, *p* < 0.001		*χ*^2^ (df = 4) = 66.694, *p* < 0.001	
North Rhine-Westphalia	44.6 (836)	55.4 (1037)	54.5 (1016)	45.5 (849)
Hesse	48.4 (319)	51.6 (340)	51.7 (344)	48.3 (321)
Lower Saxony	56.1 (736)	43.9 (576)	61.2 (805)	38.8 (511)
Berlin	51.3 (116)	48.7 (110)	67.4 (153)	32.6 (74)
Schleswig-Holstein	66.0 (443)	34.0 (228)	69.0 (461)	31.0 (207)
Total (*n* = 656)	51.7 (2450)	48.3 (2291)	58.6 (2776)	41.4 (1962)

Notes. HPS Health Promoting School, *χ*^2^ Chi Square, df degrees of freedom, % percent, *n* frequency.

**Table 5:** Multiple binary logistic regression analysis for dimensions of HPS implementation.

	HPS Implementation			
	Concrete HPS Action (Low)		HPS Capacity Building (Low)	
	OR	95% CI	OR	95% CI
Gender				
Female	1.00	-	1.00	-
Male	1.36 **	1.18–1.56	1.42 **	1.24–1.63
Age				
>60 years	1.00	-	1.00	-
46 to 60 years	1.49 **	1.26–1.76	1.15	0.97–1.36
≤45 years	1.90 **	1.55–2.34	1.47 **	1.20–1.79
Type of school				
Primary school	1.00	-	1.00	-
Secondary school	3.13 **	2.68–3.64	1.78 **	1.54–2.07
School f. child. with special educ. needs	1.00	0.80–1.23	1.29 *	1.05–1.60
Vocational school	2.20 **	1.69–2.87	0.73 *	0.56–0.94
Others	1.16	0.50–2.69	1.67	0.73–3.83
*Federal state*				
North Rhine-Westphalia	1.00	-	1.00	-
Hesse	1.15	0.94–1.40	1.00	0.82–1.20
Lower Saxony	1.45 **	1.24–1.70	1.27 **	1.08–1.48
Berlin	1.35	0.99–1.85	1.90 **	1.38–2.61
Schleswig-Holstein	2.46 **	2.01–3.01	1.95 **	1.59–2.38
Resource availability				
High	1.00	-	1.00	-
Low	1.46 **	1.27–1.67	1.29 **	1.13–1.48
Decision latitude				
High	1.00	-	1.00	-
Low	1.55 **	1.34–1.79	1.40 **	1.21–1.61
Perceived educational benefits				
High	1.00	-	1.00	-
Low	1.65 **	1.36–2.01	1.69 **	1.39–2.06

**Notes**. Concrete HPS action (*n* = 4320), HPS capacity building (*n* = 4320), Nagelkerke’s R^2^ = concrete HPS action: 0.16, HPS capacity building: 0.09; HPS: Health Promoting School, OR: Odds Ratios, CI: confidence interval, ** *p* < 0.01, * *p* < 0.05.

## Data Availability

The data presented in this study are available on reasonable request from the corresponding author. The data are not publicly available because participants did not consent to having their data made publicly available.

## Supplementary Materials

The following are available online at https://www.mdpi.com/1660-4601/18/5/2623/s1, Figure S1: Low HPS implementation stratified by federal state, Table S1: Pearson correlation, mean and standard deviation of HPS implementation (all items), Table S2: Instruments used for this survey.
