# Supplementary material for: Health Promoting Schools in Germany. Mapping the Implementation of Holistic Strategies to Tackle NCDs and Promote Health
Source: Int J Environ Res Public Health. 2021 Mar 5;18(5):2623. doi: 10.3390/ijerph18052623 (PMC7967522; doi:10.3390/ijerph18052623)
Supplement: Supplementary file 1 [file ijerph-18-02623-s001.pdf]

Table S1. Instruments used for this survey.

In the following, we ask you to make statements about your work as a school principal.
Please check the answer that most closely matches your perception.

	Not true				Completely true
I have access to the resources I need to do my job well.	☐1	☐2	☐3	☐4	☐5
I can obtain the resources necessary to support new ideas	☐1	☐2	☐3	☐4	☐5
When I need additional resources to do my job, I can usually get them	☐1	☐2	☐3	☐4	☐5

In the following, we ask you to make statements about your work as a school principal.
Please check the answer that most closely matches your perception.

	Not true at all				Completely true
Overall, the work offers many opportunities to make own decisions	☐1	☐2	☐3	☐4	☐5
I can decide for myself the way in which I do my work	☐1	☐2	☐3	☐4	☐5
I can plan my work autonomously (e.g. decide myself which material I need)	☐1	☐2	☐3	☐4	☐5
The implementation of my own ideas is difficult by the way my colleagues work	☐1	☐2	☐3	☐4	☐5

If health promotion and prevention activities were continuously implemented at my school, I believe it would have a positive impact on students' academic performance

☐1	Very unlikely
☐2	Unlikely
☐3	Likely
☐4	Very likely

In this section, we would like to know to what extent health currently plays a role at your school.
Please check the answer that most closely matches your perception.

	Strongly disagree	Rather Disagree	Rather Agree	Strongly agree
Health promotion is a topic in our school development group.	☐1	☐2	☐3	☐4
Health promotion and health goals are anchored in the mission statement and programme of our school.	☐1	☐2	☐3	☐4
At our school, health plays an important role in the organisation of lessons.	☐1	☐2	☐3	☐4
At our school, maintaining and promoting the health of all members plays an important role	☐1	☐2	☐3	☐4
Health-promoting aspects play an important role in the creation of working and learning conditions at our school.	☐1	☐2	☐3	☐4

At our school, pupils are supported in the development of health-promoting behaviours.	☐1	☐2	☐3	☐4
At our school, teachers are supported to deal with stressful situations more effectively.	☐1	☐2	☐3	☐4
Our school collaborates with external institutions in in the implementation of health-promoting activities	☐1	☐2	☐3	☐4
At our school, systematic efforts are made to improve the work situation	☐1	☐2	☐3	☐4
Our school continuously participates in projects/programs of health promotion/prevention	☐1	☐2	☐3	☐4
At our school, teachers are made aware of health-related topics such as exercise or self-management.	☐1	☐2	☐3	☐4
Further training on health-related topics take place regularly at our school.	☐1	☐2	☐3	☐4
At our school, we work closely with parents when it comes to the healthy development of children.	☐1	☐2	☐3	☐4
Health-promoting aspects are taken into account in the design of the school building and outdoor facilities (e.g., space for exercise, relaxation).	☐1	☐2	☐3	☐4

Table S2. Pearson correlation, mean and standard deviation of HPS implementation (all items).

		M (SD)	1	2	3	4	5	6	7	8	9	10	11	12	13
1	Health promotion as a topic in school development groups	2.9 (0.9)	1												
2	Health goals in the school's mission statement	2.7 (1.0)	0.58 *	1											
3	Health as a principle in the organisation of teaching	2.8 (0.8)	0.46 *	0.54 *	1										
4	Importance of health promotion for all members in the school	2.9 (0.7)	0.49 *	0.48 *	0.68 *	1									
5	Creation of health promoting working and learning conditions	2.8 (0.7)	0.44 *	0.45 *	0.63 *	0.71 *	1								
6	Promotion of healthy behaviours in students	3.1 (0.7)	0.34 *	0.41 *	0.58 *	0.54 *	0.55 *	1							
7	Support for teachers in dealing with stressful situations	2.8 (0.8)	0.39 *	0.33 *	0.46 *	0.53 *	0.52 *	0.46	1						
8	Collaboration with external stakeholders	2.4 (1.0)	0.46 *	0.45 *	0.43 *	0.42 *	0.42 *	0.39 *	0.41 *	1					
9	Systematic improvement of the work situation	2.7 (0.8)	0.37 *	0.31 *	0.38 *	0.43 *	0.45 *	0.35 *	0.48 *	0.41 *	1				
10	School participation in health promoting projects	2.3 (1.1)	0.32 *	0.38 *	0.34 *	0.30 *	0.29 *	0.32 *	0.22 *	0.46 *	0.27 *	1			
11	Awareness raising of health issues among teachers	2.6 (0.9)	0.48 *	0.44 *	0.46 *	0.46 *	0.45 *	0.42 *	0.51 *	0.49 *	0.44 *	0.40 *	1		
12	Provision of regular further training on health-related topics	2.2 (0.8)	0.48 *	0.46 *	0.43 *	0.42 *	0.41 *	0.35 *	0.44 *	0.55 *	0.41 *	0.40 *	0.62 *	1	
13	Close cooperation with parents for the healthy development of children	2.8 (0.9)	0.22 *	0.24 *	0.38 *	0.35 *	0.39 *	0.46 *	0.33 *	0.24 *	0.27 *	0.26 *	0.31 *	0.25 *	1
14	Creation of health-promoting school environment	2.7 (1.0)	0.22 *	0.26 *	0.33 *	0.33 *	0.41 *	0.35 *	0.30 *	0.25 *	0.29 *	0.22 *	0.30 *	0.27 *	0.35 *

Notes. *M* mean value, SD standard deviation, * $p < 0.001$.

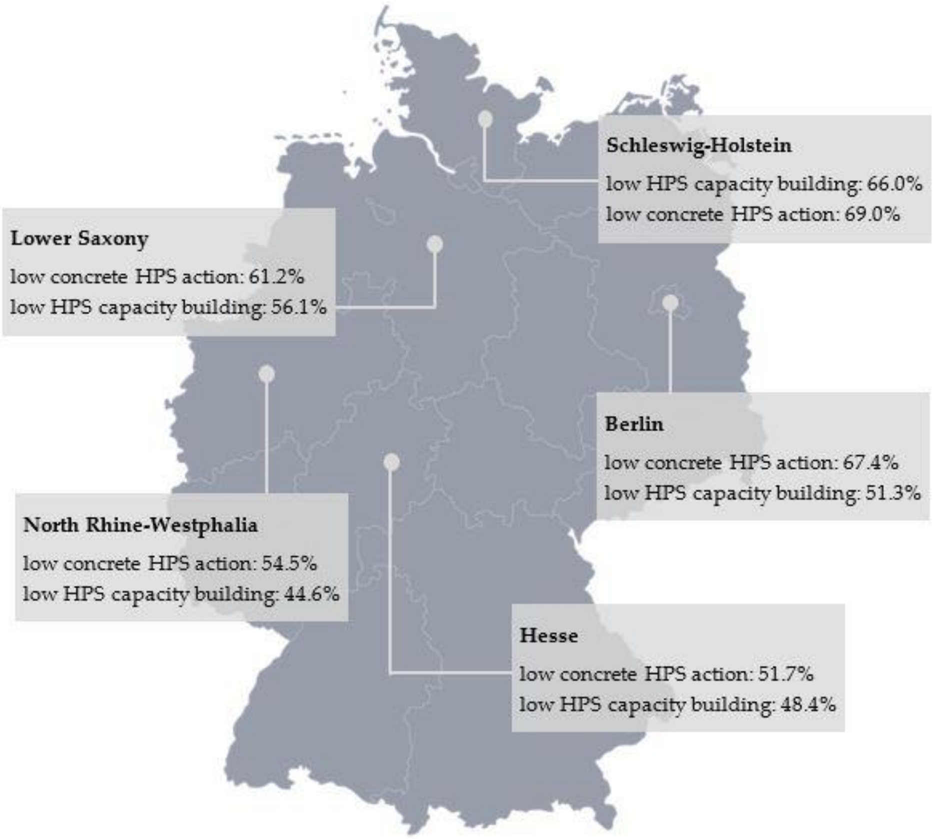

Figure S1. Low HPS implementation stratified by federal state.
